# COVID-19 Restrictions Resulted in Both Positive and Negative Effects on Digital Media Use, Mental Health, and Lifestyle Habits

**DOI:** 10.3390/ijerph20166583

**Published:** 2023-08-16

**Authors:** Sissela B. Nutley, Jonas Burén, Lisa B. Thorell

**Affiliations:** Department of Clinical Neuroscience, Karolinska Institutet, 171 77 Stockholm, Swedenlisa.thorell@ki.se (L.B.T.)

**Keywords:** COVID-19, mental health, adolescence, digital media, social media, longitudinal

## Abstract

While studies have reported effects on digital media during the COVID-19 restrictions, few have included data prior to the pandemic, and most have only measured screen time. We therefore investigated changes in specific digital media activities, as well as mental health and lifestyle habits, in a longitudinal study of adolescents spanning from before the pandemic (T1) to one month into restrictions (T2) and one year later when schools had reopened (T3). Adolescents (16–19 years) rated smartphone use, problematic/addictive media use, negative experiences (e.g., victimization), mental health (i.e., irritability, stress, and closeness), and protective lifestyle habits (i.e., sleep and exercise). Results showed initial decreases in irritability and negative digital experiences, increases in sleep and exercise, as well as a decrease in closeness during remote learning (T2). However, these changes returned to, or superseded, their initial levels at follow-up (T3). There were also increases in digital media use and stress at T3. Conclusively, by investigating specific digital media activities and collecting data both prior to and during different phases of the pandemic, we were able to find both positive and negative effects.

## 1. Introduction

Since the widespread, nearly global adoption of the smartphone about a decade ago, most adolescents and adults spend a considerable amount of their time using digital media. While technological advancements and digital media played a major role in the continuation of work and remote learning during the restrictions due to the COVID-19 pandemic, the increased time spent on digital media may also have had a negative impact on mental health [1]. The pandemic-related restrictions may have had particularly detrimental effects on children and adolescents, who rely on schools being open for their education, social connections, and maintaining daily routines [2]. Previous research conducted during the initial wave of the pandemic has shown that school closures, along with increased digital media use and changes in lifestyle habits such as lower physical activity, lead to an increase in mental health problems in adolescents [3]. However, previous research has been limited in that most studies have only investigated time spent on digital media rather than specific negative experiences (e.g., bullying, sexual harassment) or problematic use (e.g., use that is addictive and/or crowds out other important activities in life). Many studies have also used retrospective reports of changes in screen time rather than collecting data both before and during the pandemic [1,4]. To address these limitations in previous research, we collected data from adolescents on three occasions (before the pandemic, early during the pandemic, and one year into the pandemic), with our overall aim being to investigate change in digital media use (i.e., screen time, problematic use of digital media, and negative online experiences), mental health (i.e., stress, irritability, and closeness), and protective lifestyle habits (i.e., sleep and exercise) across different phases of the pandemic restrictions. 

### 1.1. Digital Media Use and Mental Health during the COVID-19 Pandemic

One of the most reported consequences of the COVID-19 restrictions has been increased screen time among adults [5,6], children and adolescents [7,8,9,10,11,12], and even toddlers [7,13]. Systematic reviews have also concluded that mental health problems (e.g., anxiety, depression, and stress) increased among adolescents during the pandemic [3,14]. However, due to the sudden onset of the pandemic restrictions, most studies have only been able to investigate the outcomes after the restrictions were imposed. This should be seen as a serious limitation, as it is well known that retrospective assessments of change in digital media use have questionable reliability (e.g., [15,16]). 

It should be noted that in addition to time spent using digital media, both the content interacted with (e.g., [17,18,19]) and the degree to which the use is problematic (or even addictive) should also be taken into consideration. In fact, recent findings suggest that problematic or addictive use of digital media is more strongly associated with mental health outcomes compared to screen time [20]. Still, very few COVID-19 studies have investigated problematic digital media use. Two studies of adolescents or college students have suggested that both problematic gaming and psychological distress have increased during the pandemic when using retrospective reports of use before the pandemic [5,21]. Furthermore, both a longitudinal study of middle school students [22] and a study using ecological momentary assessments in a group of adolescents undergoing mental health treatment reported higher problematic use of digital media during the COVID-19 restrictions compared to before [23]. The study on middle school students also found an increase in the frequency of social media checking, social media use at bedtime, social anxiety, loneliness, and depression [22] during the pandemic. Similarly, a longitudinal study of adolescents (mean age of 14) reported an increase in mental health problems (e.g., symptoms of anxiety, depression) along with increased social media use from pre-pandemic to lockdown one year later [9]. 

### 1.2. Negative Online Experiences during the Pandemic

As a result of the characteristics associated with social interaction via screens, studies have shown that the lack of immediate social feedback (e.g., eye contact) can lead to so called online disinhibition with harsher interactions compared to those that would be considered acceptable in face-to-face interactions [24,25]. In studies conducted before the pandemic, psychological distress has been linked to specific negative online experiences such as online bullying [17,26], sexual harassment [18,27], and being exposed to images depicting beauty ideals [19,28,29]. Sexting has also been shown to be associated with mental health difficulties in adolescents [30]. 

With increased time spent online during the pandemic restrictions, one could expect that the prevalence of negative online experiences would also increase. It is also possible that online disinhibition would increase when adolescents knew that they would most likely not meet their peers in face-to-face interactions for an extended period due to school closures. Interestingly, one study showed support for the opposite, with a decrease in all forms of bullying during remote learning using retrospective accounts [31], while a longitudinal study did not detect any change in online bullying at all during the pandemic [22]. To our knowledge, there are no studies of adolescents that have assessed issues related to one’s self-image or reputation on social media or sexual harassment during the COVID-19 pandemic using a longitudinal design.

### 1.3. Lifestyle Habits during the Pandemic

Other areas likely impacted by the pandemic restrictions are lifestyle habits such as sleep and physical activity. Studies conducted before the pandemic have shown that sleep and physical activity are important protective factors for mental health [32,33], and it is also well known that too much time spent on digital media has negative effects on both sleep [34] and exercise [35]. Interestingly, initial reports suggest that the COVID-19-related restrictions had a differential impact on sleep and exercise. For example, a longitudinal study reported that the proportion of adolescents meeting the sleep recommendations of 8 h/night increased from 13.4% to 37.5% [36]. However, for physical activity, a decrease has been reported in many countries [37]). One plausible explanation for this is that, during the pandemic-related school closures, many children could sleep longer because they did not have to travel back and forth to school. On the other hand, they missed out on their daily exercise by having to walk/cycle to school or participating in physical education as part of the school curriculum. This is of importance, as one review of mental health during the COVID-19 pandemic concluded that receiving social support and getting exercise regularly were particularly protective for mental health [38].

### 1.4. Sex Differences in Digital Media, Lifestyle Habits and Mental Health during the Pandemic

Female adolescents typically suffer from worse mental health than males [39], and it has been suggested that the pandemic restrictions had particularly negative effects on females [14]. In addition, a study found that females reported higher levels of negative online experiences and loneliness than males during the pandemic [40]. Pre-pandemic studies also show that females are more often subjected to sexual harassment on social media than males [18,27,41]. A study collecting retrospective reports from a large sample of young Spanish females (14–24 years of age) reported an increase in the number of appearance-related accounts from pre-pandemic to lockdown, which was related to an increase in body dissatisfaction [42].

In summary, while mainly correlational studies have concluded that the pandemic restrictions largely had a negative impact on adolescent mental health, there are also reports indicating that some aspects may have been positive, at least for some. To advance our understanding of the support adolescents may need stemming from pandemic restrictions, we need to assess the impact on digital media use, lifestyle habits, and mental health issues.

### 1.5. Aims of the Present Study

The onset of the COVID-19 pandemic was very sudden. For this reason, studies investigating the effects of the pandemic on adolescents’ digital media use often lack pre-pandemic data. In addition, most studies have only included measures of time spent using digital media rather than also examining problematic use or specific online activities. The data for the present study was collected before the pandemic as part of another study, which allowed us to track COVID-19-related impacts from the time before restrictions and across two time points during the pandemic. To add to the previous research, the primary aim of the present study was to investigate the impact of the COVID-19 pandemic on (1) problematic digital media use, (2) negative online experiences (i.e., victimization, concerns about one’s appearance on social media, sexual harassment), (3) mental health, and (4) protective lifestyle habits (i.e., sleep and exercise). A secondary aim was to investigate sex differences regarding effects across different phases of the pandemic. 

## 2. Materials and Method

### 2.1. Participants and Procedure

**Figure 1:**
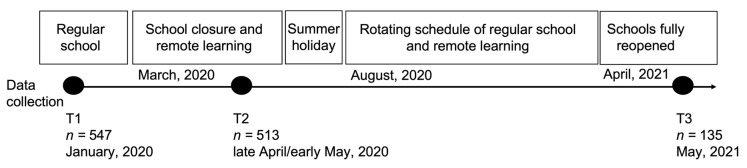
Timeline with important dates pertaining to the pandemic restrictions in Sweden, with black dots denoting the points of data collection.

The participants were recruited to the study by request from a public high school in central Sweden as part of a school-run mental health initiative that included a lecture on lifestyle and digital media habits and mental health for the entire school. All students at the school (approximately 650) were invited to participate in the study. After providing informed written consent, the students received a digital link where they were asked to complete an anonymous survey in class, pre-pandemic in January 2020 (T1 = pre-COVID-19; see Figure 1 for a timeline). No support was required by the researchers or teachers during the data collection. This resulted in a total of 547 students (68.2% females, age range 15–19, *M* = 16.93, *SD* = 0.83, 8.6% with an immigrant background) who completed the survey at baseline. The school then switched to remote learning when all high schools in Sweden closed in the middle of March 2020, and we therefore contacted the school again as we saw an opportunity to investigate the effects of the COVID-19 pandemic restrictions. In a second wave, data were collected in late April/early May 2020 (T2 = 1–2 months into pandemic restrictions), resulting in 513 students (68.6% females) who were manually matched to responses collected at T1 using birthdate and sex. The remote learning continued during the Autumn term until mid-November. The schools then gradually increased regular classroom sessions on a rotating schedule, resulting in, on average, one or two days at school per week for the students. Due to a surge in the spread of the virus, the schools were forced again to conduct remote learning for two weeks during January, after which the rotating schedule was used until the schools re-opened full-time in April 2021. In the final wave, data were collected in May 2021 (T3 = 14 months after the beginning of restrictions), with 166 respondents. Only students who completed at least two assessments were included in the analysis. At T3, this resulted in 135 matched (to T1 or T2) students, bringing the final sample included in the mixed model analysis to T1: 547; T2: 513; and T3: 135. The relatively low response rate for T3 was mainly a result of the fact that the seniors from previous waves had graduated, and because we did not have their contact information, we could not reach them at T3. The proportion of students with an immigrant background was similar across all time points (ranging between 8.6 and 9.6%). The sample at T2 did not differ significantly from the sample at T1 regarding sex or age. However, the proportion of females participating at T3 was significantly lower (56.3% females) than among T1 and T2 only responders (71.9 and 71.1%), *χ*^2^ > 6.5, *p* < 0.02). The sample responding at T3 did not differ on any of the other outcomes at T1 compared to non-responders at T3 (*t* values between −0.17 and 1.23, all *p*s *>* 0.19). The study was carried out in accordance with the Code of Ethics of the World Medical Association (Declaration of Helsinki) for experiments involving humans. 

### 2.2. Materials

All outcomes were measured on a 5-point scale ranging from 0 to 4, with higher scores indicating more frequent or more severe symptoms. 

#### 2.2.1. Digital Media Use

Digital media use was assessed through measures of time spent on mobile phones and problematic use of digital media. For the time variable, we asked how many hours the participants spent on their mobile phones on a typical weekday for leisure purposes (excluding school related use), using the following response scale: 0 = <1 h, 1 = 1–2 h, 2 = 2–4 h, 3 = 4–6 h, 4 = >6 h. Problematic use of digital media was assessed using the mean score for responses to four statements rated on a Likert scale ranging from 0 (strongly disagree) to 4 (strongly agree). The statements were as follows: my digital media use (1) crowds out chores or basic needs (e.g., sleep, schoolwork, exercise), (2) is taking more time than I like, (3) is strongly addictive, and (4) is something I have tried to reduce without success (*α* = 0.71). 

#### 2.2.2. Negative Online Experiences

Three aspects of negative online experiences were assessed by asking the participants how often in the past month they had (1) been victimized online (“subjected to mean comments or been bullied in games or social media”), (2) worried about social media (mean score of the two items “felt pressure to look good on social media” and “worried that something negative about them would go viral on social media”; Spearman’s *ρ* = 0.53), and (3) had negative online sexual experiences (mean score of the two items “had nudes sent to you against your will” and “been asked to send nudes against your will”; Spearman’s *ρ* = 0.78). The following response scale was used: 0 (never), 1 (not more than once a week), 2 (twice a week), 3 (at least three times a week), and 4 (every day). 

#### 2.2.3. Mental Health-Related Outcomes

Three mental health-related outcomes were assessed: self-perceived irritability, stress, and closeness. These outcomes were assessed by asking how often, during the past month, “have you had irritability or a bad temper?” “have you felt stressed?” and “have you felt close to other people?” Responses were rated on the following scale: 0 (never), 1 (not more than once a week), 2 (twice a week), 3 (at least three times a week), and 4 (every day). 

#### 2.2.4. Lifestyle Habits

The protective lifestyle habits assessed were exercise and sleep. For exercise, we asked how often participants exercised for at least 40 min a day. The following response scale was used: 0 (never), 1 (not more than once a week), 2 (twice a week), 3 (at least three times a week), and 4 (every day). For sleep, we asked how many hours they slept on a normal weekday. The responses were made on the following scale: 0 = <6 h, 1 = 6–7 h, 2 = 7–8 h, 3 = 8–9 h, and 4 = >9 h. 

### 2.3. Data Analyses 

All data analyses were conducted using SPSS, version 26.0. The outcome variables were analyzed using a linear mixed-effects model fitted with full information and maximum likelihood estimation [43]. The modeling approach utilized all available data from all participants, with responses from at least two time points. As mixed-effects models offer greater flexibility in dealing with complex data structures compared with traditional statistical methods, they are considered the preferred choice for longitudinal data analyses [44]. For each dependent variable, the baseline model only included the intercept, and more complex models were tested by stepwise addition of the following variables: time as a repeated fixed factor (coded categorically), sex as a fixed factor, the interaction term between the two fixed factors (i.e., time x sex), age as a covariate, and individual random slopes. Models were also run with time as a quadratic term, which did not improve model fit. Model selection was determined by means of goodness of fit statistics (−2 log likelihood ratio tests). For the model with mobile screen time as the dependent variable, age significantly improved the model fit and was thus included as a covariate. For the models with problematic use, exercise, and sleep, a random intercept (scaled identity covariance structure) improved model fit. The robustness of the results was ensured with sensitivity analyses by running the linear mixed models using a random subset with 80% of the sample as well as by running linear regression models structuring the data in wide format with either T2 or T3 as the dependent variable and T2 or T1, respectively, as independent variables along with sex and age (resulting in listwise deletion of missing data). However, these alternative methods of analyzing the data did not change the main results. Reliability of two item scales is reported with the Spearman-Brown coefficient (*ρ*) [45], where *ρ* values between 0.5 and 0.7 are considered fair, 0.7–0.9 are considered good, and above 0.9 is considered excellent [46]. Reliability for scales consisting of more items is reported using Cronbach’s alpha (*α*), where values between 0.7 and 0.95 are generally considered acceptable [47]. 

## 3. Results

### 3.1. Pandemic Related Effects on Digital Media Use

The parameter estimates from the final models are reported in Table 1. For digital media use, time spent on mobile phones for leisure purposes increased substantially throughout the course of the study for both sexes and was consistently higher for females than for males. 

**Figure 2:**
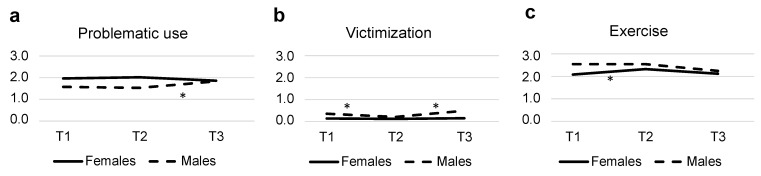
Parameter estimates for the significant interaction terms between sex and time from the mixed models reported in Table 1. (**a**) problematic use of digital media; (**b**) victimization; (**c**) exercise. * = indicates for what time point (i.e., T1 vs. T2 or T2 vs. T3) that the interaction effect is significant.

Females also showed higher rates of problematic digital media use. However, a significant interaction effect between time and sex for problematic digital media use was also found, which indicated an increase from T2 to T3 for males but not for females, bringing males up to the same level as females regarding problematic use of digital media at T3 (see Figure 2a).

### 3.2. Specific Negative Digital Media Experiences

Regarding negative online experiences, there were significant declines between T1 and T2 for both social media worry and online victimization. However, the decline for unwillingly receiving or being asked to send nudes was not statistically significant. To exemplify the magnitude of these effects for the entire sample, we calculated the frequency of adolescents having had these experiences at least once in the past month across the three time points. Results indicated that from T1 to T2, there was a 19% decline in being victimized (T1: 16%, T2: 13%, T3: 19%), a 17% decline in being worried about appearance on social media (T1: 66%, T2: 55%, T3: 56%), and a 21% decline in unwillingly being asked to send or receive nudes (T1: 38%, T2: 30%, T3: 30%).

Regarding sex differences, females were twice as often worried about social media-related issues compared to males, and they were also twice as often subjected to receiving or being asked to send nudes (see Table 1). Males reported being victimized more often compared to females. For online victimization, males showed a decrease from T1 to T2 and then a return to even higher levels at T3, whereas females reported the same level of online victimization throughout the study (see Figure 2b). 

### 3.3. Mental Health

The results for mental health showed a significant effect of time on both stress and closeness. More specifically, there was a decline in feeling close to others at T2 when schools switched to remote learning, which then returned to initial levels when schools reopened at T3. Stress, on the other hand, did not show a significant change from T1 to T2, but increased significantly between T2 and T3. Irritability showed a slight decrease between T1 and T2, which was not statistically significant. Compared to males, females reported higher levels across all time points for both stress and irritability; however, no sex difference was found for closeness. There were no significant interactions between time and sex for any of the mental health measures.

### 3.4. Lifestyle Habits

There was also evidence of significant changes in lifestyle habits throughout the course of the study, with females showing an increase in exercise at T2 (see Figure 2c), approaching the males’ level of exercise. There was also a decline in exercise for both sexes between T2 and T3. Generally, males reported higher levels of exercise compared to females. Regarding sleep, there was an increase in sleep between T1 and T2, followed by a return to initial levels at T3 for both males and females. No significant sex difference was found for sleep, and there was no evidence of significant interaction effects between time and sex for either sleep or exercise. 

## 4. Discussion

The main findings from the present study were that multiple areas of young people’s lives underwent significant changes during the pandemic restrictions. Effects ranged from digital media use and negative online experiences to mental health outcomes and lifestyle habits. The study confirmed that females reported more problems with mental health, social media worry, and being sexually harassed across all time points compared to males, while males were more often subjected to online bullying. Furthermore, we report several changes in specific aspects of digital media use during the pandemic. First, although time spent using mobile phones increased substantially over time for both sexes, problematic use of digital media did not increase immediately during remote learning but did increase slightly one year later for boys. Second, we showed that negative online experiences declined during remote learning, only to return to or even supersede initial levels one year later. Mental health problems and lifestyle habits also showed significant changes during the COVID-19 pandemic. The negative effects observed were, not surprisingly, a decrease in closeness during remote learning and an increase in stress when schools reopened. Positive effects included increased levels of sleep and exercise and decreased levels of irritability. 

### 4.1. Negative Effects of the Pandemic

Our finding related to increased time spent using mobile phones for leisure purposes during the remote learning phase is consistent with results from previous studies [9,10,11,12,22] and extends these results by showing that this increase continued even as schools reopened, 14 months after the initial restrictions. This increased time spent on mobile phones throughout the first year of the pandemic brought both sexes up to an average of 4–6 h/day. Given that previous studies have established an increased risk of mental health issues with use of more than 2–3 h/day [48,49], this magnitude of use as the ‘new normal’ warrants concern. Recent reviews have concluded that the increased use of social media during the pandemic is associated with the increased mental health issues reported in youth [1,4]. Moreover, at the same time, it is apparent that social media are also important platforms for receiving social support [50].

It is also important to note that while several other outcomes showed a u-shaped distribution across time (i.e., a return to baseline levels at T3), adolescents reported increased levels of stress when schools reopened. Considering that previous studies have demonstrated a significant decline in academic progression due to remote learning [51,52], it is possible that the increase in stress observed in our study is at least partially a result of the cumulative strain of struggling academically during remote learning. While the relations between digital media use and mental health outcomes were not examined in this study due to a lack of statistical power at T3, previous studies report the use of digital media as a coping strategy to combat increased stress [5,53]. While using digital media may serve this purpose temporarily, other findings [54] suggest that problematic use of and/or increasing time spent on digital media may in fact exacerbate stress and mental health problems over time, especially for females. This highlights the importance of conducting further follow-ups to investigate more long-term negative consequences of the pandemic. 

### 4.2. Positive Effects of the Pandemic

Interestingly, although the time spent on digital media increased, two types of negative online experiences investigated in the present study (i.e. social media worry and victimization) decreased from T1 to T2. Few previous COVID-19 studies have investigated this issue. Our findings are in contrast with retrospective reports on females showing an increase in appearance-based concerns during the lockdown [42]; however, in line with the retrospective reports by Vailliancourt et al. showing a decline in online bullying during school closures [31]. The longitudinal study by Charmaraman et al. did not detect any change in online bullying during the pandemic [22]. Unfortunately, our study showed that all declines in negative online experiences returned to, or even surpassed, their initial levels when schools reopened (i.e., T3). The temporary declines during remote learning indicate that the lack of physical interaction with peers led to fewer instances that previously would have triggered these negative online experiences. This pattern of change over time suggests that these effects were most likely due to the school closures rather than to a general effect of age. These findings support the notion that online victimization and face-to-face victimization in the school setting should be regarded as two sides of the same coin rather than as two separate phenomena [55]. 

Negative online experiences are of great concern because they are known to be related to anxiety, low self-esteem, and depression [17,26,29]. The general decline in, for example, victimization found in the present study could be regarded as small. However, because many adolescents did not encounter negative online experiences, mean values may not provide the best representation of the effects. For this reason, we also reported the frequency of these events during the past month and were able to show that negative experiences during remote learning declined between 17% and 21%. It should also be noted that the proportion of adolescents reporting negative online encounters at least once during the past month was remarkably high (i.e., ranging between 16 and 66% at T1). Our findings are in line with previous studies [18,40] showing that females consistently report more negative online encounters (e.g., receiving unrequested “dick picks” and other forms of sexual harassment) compared to males. Although pandemic effects differed slightly between males and females (e.g., decreased victimization only for males), our results did not support those of previous studies showing that the pandemic effects were generally worse for females [7,12].

Regarding lifestyle habits, the pandemic-related restrictions seem to have increased sleep, which is in line with previous findings [36]. This may have been an important factor in mitigating mental health issues, as sleep deprivation during adolescence is associated with an increased risk of developing anxiety and depression over time [56,57]. It should also be noted that adolescents’ sleep time increased during school closures even though their time on mobile phones also increased. This is not in line with studies conducted before the pandemic, which have shown that higher screen time is associated with lower amounts of sleep [34]. However, due to, for example, delayed school start times, no traveling to and from school, and the cancellation of extra-curricular activities, many adolescents had the time to use digital media more during the pandemic without it interfering with their sleep. An additional explanation for the decrease seen in victimization could be that cyberbullying perpetrators also increased their sleep, making them less inclined to bully others, as a previous study reported that lack of sleep explained cyberbullying through increased anger [58].

In contrast to previous findings [37], we found that exercise also increased during remote learning. However, the increase in exercise was only seen in females, possibly because girls took more walks together to socialize with their friends, whereas males were limited by the fact that gyms were closed and organized team sports activities were canceled. It is interesting to note that whereas research conducted before the pandemic has shown that digital media use leads to the displacement of health promoting activities such as sleep and exercise [59], our study found an increase in both digital media use and health promoting activities. This is most likely because the time spent in class, socializing with friends, and traveling to and from school decreased during the pandemic, leaving more time to use digital media as well as exercise and sleep. 

### 4.3. Strengths and Limitations

Regarding strengths, the present study is, to our knowledge, the first to measure specific negative online experiences among adolescents using a longitudinal design that covers the period from before the pandemic, across the early pandemic phase, and one year later when schools reopened. This should be considered particularly beneficial, as retrospective ratings of digital media use and mental health have questionable reliability. This is also the first study to track specific online experiences rather than merely investigating time spent online, which has furthered our understanding of how digital media use has been influenced during the pandemic. This natural experiment has also given us further insight into how the school environment may act as a catalyzing platform, transferring offline victimization to an online setting, which was mitigated when schools were closed. 

Some limitations should also be noted. First, the retention rate at T3 was low, limiting the possibility of assessing possible changes in the relationships between the outcomes over time. Although a large part of the attrition was caused by the fact that about one third of the sample graduated between T2 and T3, some students simply did not respond to the final questionnaire. Communication with the school revealed that the previous extra burden of logistics caused by the rotating remote learning schedules and the stress of not having made enough academic progress resulted in fewer teachers taking the time out of class for the students to respond to the questionnaire at T3. However, as explained in the method section, sensitivity analyses suggest that the results are nonetheless robust. Second, several of the outcomes were based on single- or two-item responses. While this is suboptimal, it was necessary to keep the student questionnaires very short, as they were completed in class. It also enabled us to obtain a high response rate, with practically all students who were present in school on the day of the survey choosing to participate. This should be considered a strength of the present study, as the importance of including vulnerable subgroups in pandemic-related research has been emphasized [60]. Third, the present study only addressed subgroups based on sex, while other studies have demonstrated that the school closures have had a particularly great impact on vulnerable subgroups, such as children with mental health problems [1,61] or special educational needs [62,63]. Previous research has also shown that parental mediation has been important for children’s digital media use during the COVID-19 pandemic [64], which suggests that children have also been differently affected depending on parenting related aspects. Unfortunately, the sample size of the present study was too small to allow investigation of effects in subgroups that might have been especially vulnerable to the effects of remote learning during the COVID-19 pandemic. Finally, although the present study included a few measures of social media content rather than just focusing on screen time, it would have been valuable to include a broader range of both positive and negative content (e.g., support groups, unhealthy beauty ideals, health information about COVID-19).

## 5. Conclusions

The restrictions caused by the COVID-19 pandemic seem to have had short-term positive effects, as seen in decreases in negative experiences online and irritability, along with more sleep and exercise. However, the consequences one year later point to increased stress and time spent on mobile phones and a return to baseline levels for both negative experiences on social media and lifestyle habits (i.e., sleep and exercise). The implications of the temporary decreases in these negative experiences due to remote schooling warrant new ways of thinking to prevent victimization, sexual harassment, and worry about social media appearances. Previous studies have demonstrated that, when young people need support, these types of encounters may be difficult to communicate with adults [65]. Our findings, as well as those of previous studies, should motivate general classroom interventions both to prevent bullying and harassment, promote establishing norms for a better digital climate, and teach mental health strategies. Furthermore, this study highlights the importance of parents supporting adolescents in moderating both the content and time spent on digital media so that priority is given to forming lifestyle habits that will promote mental health. 

## Figures and Tables

**Table 1:** Parameter estimates (and 95% confidence intervals) from the mixed models with respective outcomes as dependent variables, sex and time as fixed factors, and an interaction term between the two (T × S).

Outcome	T1	T2	T3	*F* Time	*F* Sex	*F* T × S	Post Hoc T
**Digital media use**							
Mobile time M	1.99 (1.86–2.12)	2.17 (2.04–2.30)	3.00 (2.78–3.22)	58.48 ***	16.18 *** F > M	0.69	T1 < T2 < T3
F	2.32 (2.23–2.41)	2.52 (2.43–2.61)	3.18 (2.97–3.37)				
Problematic use M	1.57 (1.44–1.70)	1.53 (1.40–1.67)	1.83 (1.63–2.04)	0.67	15.87 *** F > M	5.40 **	
F	1.96 (1.87–2.05)	2.02 (1.92–2.11)	1.86 (1.68–2.03)				
**Negative Digital media experience**							
Social media worry M	0.33 (0.21–0.44)	0.27 (0.15–0.38)	0.40 (0.21–0.59)	7.33 **	60.75 *** F > M	2.39	T1 > T2 < T3
F	0.92 (0.84–1.00)	0.71 (0.63–0.79)	0.84 (0.68–1.01)				
Nudes M	0.17 (0.09–0.23)	0.13 (0.06–0.21)	0.20 (0.09–0.32)	2.56	18.30 *** F > M	1.53	
F	0.39 (0.34–0.44)	0.30 (0.26–0.35)	0.29 (0.19–0.39)				
Victimization M	0.36 (0.27–0.44)	0.21 (0.12–0.29)	0.49 (0.35–0.62)	6.45 **	24.71 *** M > F	4.43 **	T1 > T2 < T3
F	0.14 (0.08–0.19)	0.12 (0.06–0.18)	0.15 (0.03–0.27)				
**Mental health**							
Irritability M	1.82 (1.66–1.98)	1.71 (1.55–1.87)	1.65 (1.39–1.90)	2.32	14.82 *** F > M	0.41	
F	2.10 (1.99–2.21)	1.98 (1.87–2.09)	2.09 (1.86–2.31)				
Stress M	1.92 (1.74–2.11)	1.96 (1.78–2.15)	2.36 (2.06–2.65)	5.19 **	37.98 *** F > M	1.68	T1, T2 <T3
F	2.71 (2.58–2.83)	2.55 (2.42–2.67)	2.83 (2.57–3.09)				
Closeness M	2.83 (2.64–3.02)	2.31 (2.12–2.50)	2.76 (2.45–3.06)	19.78 ***	2.36	1.72	T1 > T2 < T3
F	2.87 (2.75–3.00)	2.59 (2.47–2.72)	2.88 (2.62–3.15)				
**Lifestyle habits**							
Exercise M	2.54 (2.37–2.71)	2.54 (2.37–2.71)	2.24 (1.99–2.50)	5.59 **	8.01 ** M > F	3.59 *	T1 < T2 > T3
F	2.08 (1.96–2.20)	2.32 (2.20–2.43)	2.11 (1.89–2.33)				
Sleep M	1.43 (1.29–1.58)	1.80 (1.67–1.94)	1.41 (1.21–1.62)	40.52 ***	2.16	0.35	T1 < T2 > T3
F	1.53 (1.42–1.63)	1.96 (1.88–2.05)	1.48 (1.30–1.66)				

M = males, F = Females. * *p* < 0.05, ** *p* < 0.01, *** *p* < 0.001.

## Data Availability

The datasets generated and/or analyzed during the current study are not publicly available but are available from the corresponding author upon reasonable request.
